# In Silico Studies on Compounds Derived from *Calceolaria*: Phenylethanoid Glycosides as Potential Multitarget Inhibitors for the Development of Pesticides

**DOI:** 10.3390/biom8040121

**Published:** 2018-10-23

**Authors:** Marco A. Loza-Mejía, Juan Rodrigo Salazar, Juan Francisco Sánchez-Tejeda

**Affiliations:** Benjamín Franklin 45, Cuauhtémoc, Mexico City 06140, Mexico; juansanchez@lasallistas.org

**Keywords:** molecular docking, bioinsecticides, structure–activity relationship, phenylethanoid glycosides, *Calceolaria*, multitarget

## Abstract

An increasing occurrence of resistance in insect pests and high mammal toxicity exhibited by common pesticides increase the need for new alternative molecules. Among these alternatives, bioinsecticides are considered to be environmentally friendly and safer than synthetic insecticides. Particularly, plant extracts have shown great potential in laboratory conditions. However, the lack of studies that confirm their mechanisms of action diminishes their potential applications on a large scale. Previously, we have reported the insect growth regulator and insecticidal activities of secondary metabolites isolated from plants of the *Calceolaria* genus. Herein, we report an in silico study of compounds isolated from *Calceolaria* against acetylcholinesterase, prophenoloxidase, and ecdysone receptor. The molecular docking results are consistent with the previously reported experimental results, which were obtained during the bioevaluation of *Calceolaria* extracts. Among the compounds, phenylethanoid glycosides, such as verbascoside, exhibited good theoretical affinity to all the analyzed targets. In light of these results, we developed an index to evaluate potential multitarget insecticides based on docking scores.

## 1. Introduction

The continuous growth of the world population has created an enormous pressure to satisfy the global demand for agricultural products. The challenges include the depletion of soil fertility, the constant depredation of natural soils to convert them into agricultural ecosystems, and the ability of the arthropods to obtain resistance against traditional insecticidal controls.

Insecticides have been used for combating insect pests, mainly to increase the yield of food production among other agricultural products. From ancient times, there are records that describe the use of different types of products to combat insect pests [1]. It is known that various nonspecific agents have been used, such as sulfur and poisonous natural extracts, then organochlorines, organophosphates, carbamates, pyrethroids, and rotenoids, among others, and finally compounds, which are specifically designed and synthesized against enzymatic systems of arthropods.

The increasing need for agricultural goods has resulted in misutilization of insecticides, and this has led to the use of a higher concentration of insecticides or to the need for more toxic products. This has resulted in increased toxic effects on other beneficial organisms that coexist with pests in agroecosystems and on the bioaccumulation of higher concentrations of toxic insecticides in the bodies of predators or the final consumers, including humans. Despite these problems, the use of insecticides is needed to satisfy global demand for products. Thus, we can say that insecticides are a necessary evil. However, research must be carried out to identify better alternatives.

Among these alternatives, bioinsecticides enjoy a good reputation and are generally regarded as environmentally friendly and safer than synthetic insecticides [2]. For some years, many groups have conducted studies for biodirected phytochemical screening on plants toward the isolation and characterization of extracts and compounds that are useful as biocides. In most of published reports, authors investigate the effect of extracts or compounds against specific pest organisms, or against one or more isolated molecular targets, such as acetylcholinesterase (AChE) [3]. We use the extraordinary ability of plants to respond dynamically to herbivory through several molecular mechanisms, including the biosynthesis of defensive compounds to identify those with potential to be used for pest control. Those compounds can affect feeding, growth, and survival of insects and are widely distributed in nature [4]. The organic extracts prepared from the botanical material are a rich source of many classes of secondary metabolites. Many of them have been isolated via traditional chromatographic techniques and even used as active components in botanical pest management products, mainly rotenone, nicotine, strychnine, neem extracts, and essential oils [5]. Thus, the traditional methodology to discover new insecticides includes phytochemical work for the screening of microbial metabolites, terrestrial plants, algae, marine organisms, and so forth. Several factors make harder or more complicated the transition from synthetic insecticides to bioinsecticides. Specifically, the use of an extract generally does not offer guarantees of success in combating pests, and often, the insecticidal mechanisms involved are unknown or are too difficult to elucidate due to the complexity of mixtures of natural extracts [6].

However, less emphasis is given to pesticide-discovery efforts based upon natural products as templates for new structures via semisynthesis. In recent years, a renewed interest in obtaining biologically active compounds from natural sources has emerged, not only as a source of new molecules but also with innovative methodologies, including fragment-based design, high-throughput screening, and genetic engineering, towards the development of new pest-management products with low or absent toxicity towards nontarget insects and mammal organisms, low final concentrations caused by ambient degradability, and a relatively low cost compared with those compounds obtained via complete chemical synthesis [7]. 

Our group has conducted studies on the *Calceolaria* genus for the identification, isolation, and characterization of new bioinsecticides. The extracts and several secondary metabolites isolated from *Calceolaria* exhibit insect growth regulator (IGR) or insecticidal activities. The insecticidal activity was assayed against the fruit fly *(Drosophila melanogaster*, Diptera), yellow meal worm (*Tenebrio molitor*, Coleoptera: Tenebrionidae), and fall armyworm (*Spodoptera frugiperda*, J. E. Smith, Lepidoptera: Noctuidae), which are important insect pests in fruits, stored grains, and corn [8]. The experimental results indicate that some extracts and compounds isolated from *Calceolaria* interfere with sclerotization and molting processes, suggesting interaction with an ecdysone receptor [9]. Several of these extracts and compounds also act as enzymatic inhibitors against tyrosinase and protease enzymes [10], suggesting potential multitarget activity. Few examples of multitarget insecticides have been reported in the literature [11,12].

On the other hand, among the strategies used to find bioactive candidates, structure-based virtual screening (SBVS) has played a critical role, especially in the identification of potential chemotypes [13]. Docking-based virtual screening (DBVS) is probably the most widely used of these strategies. It involves docking of a library of ligands into a biological target and estimating the probability that a ligand will bind to the protein target by the application of a scoring algorithm, aiding in the identification of the most promising lead compounds for biological assays [14]. However, DBVS has some limitations: (a) the content and quality of the compound library has a profound effect on the success of DBVS, thus it is important to filter the library using the rule-of-five or other physicochemical filters, and (b) with the actual scoring functions, the prediction of correct binding poses is feasible but high accuracy prediction of binding affinity is still a challenge, thus there is little confidence on docking scores to rank potential ligands, particularly on those of the same structural frame [14,15]. Despite these limitations, DBVS has been successfully used in the identification of potential templates for new drug development [16,17,18]. Recently, some examples of the use of virtual screening and other computational chemistry tools in natural products research have been described [19,20,21,22].

With this in mind, we wanted to determine the potential use of compounds isolated from *Calceloraia* as leads in the discovery of multitarget insecticides using DBVS on some proteins recognized as targets for pesticides [12,23,24] and that could be targets for compounds present in *Calceolaria* extracts based on experimental results [9,25,26]: acetylcholinesterase (AChE), prophenoloxidase (PPO), and ecdysone receptor (EcR). Construction of a ligand library was based on compounds isolated and present in organic extracts with experimentally demonstrated pesticide activity aiming to identify potential structural templates that could be used in the development of new pesticides.

## 2. Materials and Methods

### 2.1. Ligand Construction

All of the ligands were chosen from a previously published review [27], which includes several compounds from different chemical families, including diterpenes, triterpenes, and naphthoquinones with a potential pesticidal activity, and some bioactive flavonoids and phenylethanoid glycosides as well. All of the structures were constructed using Spartan ’10 for Windows, and these geometries were optimized using the MMFF force field. Then, these structures were exported to Molegro Virtual Docker 6.0.1 [28]; assignments of charges and ionization were based on standard templates as part of the Molegro software. A complete list of all ligands and their structures are presented in Supplementary Information.

### 2.2. Molecular Docking Studies

The docking studies were carried out based on the crystal structures of *Drosophila melanogaster* acetylcholinesterase (*Dm*AchE, PDB codes: 1DX4 [29] and 1QON [29]), *Heliothis virescens* ecdysone receptor (EcR, PDB code: 3IXP [30] and 2R40 [31]), and *Manduca sexta* prophenoloxidase (PPO, PDB code: 3HHS [32]). Two different structures from the Protein Data Bank (PDB) were selected to analyze repeatability of results, independent of the PDB structure selected. This was not possible for PPO as no other PDB structure has been reported. In addition, docking studies were carried out in human acetylcholinesterase (*h*AChE, PDB code: 4EY7 [33] and 4M0E [34]) to determine whether some of the compounds exhibited theoretical preference to the *Drosophila/*human enzyme. All structures were retrieved from the Protein Data Bank [35]. Docking studies were carried out using a previously reported methodology [36,37]. Briefly, all of the solvent molecules and cocrystallized ligands were removed from the structures. Molecular docking calculations for all of the compounds with each of the proteins were performed using Molegro Virtual Docker v. 6.0.1 [28]. Active sites of each enzyme were chosen as the binding sites and delimited with a 15 Å radius sphere centered on the cocrystallized ligand, except for the PPO structure, which has no cocrystallized ligand, and the sphere was centered on the active Cu^2+^ ions. Standard software procedure was used. The assignments of charges on each protein were based on standard templates as part of the Molegro Virtual Docker program, and no other charges were necessary to be set. The Root Mean Square Deviation (RMSD) threshold for multiple cluster poses was set to <1.00 Å. The docking algorithm was set to 5000 maximum iterations with a simplex evolution population size of 50 and a minimum of 25 runs for each ligand. After docking, *MolDock Score* was calculated as the theoretical binding affinity. To assess the efficacy of this procedure, cocrystallized ligands were also docked to their respective receptors (except for PPO), the top-ranking score was recorded, and the RMSD of that pose from the corresponding crystal coordinates was computed. In all the cases, the RMSD was lower than 2 Å. For each enzyme, the 10 compounds with lower *MolDock scores* were selected for analyzing their docking poses to identify potential structural requirements for enzyme binding.

### 2.3. Molecular Dynamics Simulations

Molecular Dynamics (MD) simulations were carried out to observe differences that could account for potential selectivity of phenylpropanoids for *Dm*AChE over *h*AChE. Simulations were performed in YASARA Dynamics v.18.4.24 [38,39] using AMBER14 force field [40]. The initial structures for the MD simulation were obtained from the docking complexes of compound **87** with *Dm*AChE (PDB code: 1QON) and with *h*AChE (PDB code: 4M0E). Compound **87** (verbascoside) was selected, as it is the most studied compound of the phenylpropanoids derived from *Calceolaria*. Each complex was positioned into a water box with a size of 100 Å × 100 Å × 100 Å, with periodic boundary conditions. Temperature was set at 298 K, water density to 0.997 g/cm^3^, and pH to 7.4. Sodium (Na^+^) and chlorine (Cl^−^) ions were included to provide conditions that simulate a physiological solution (NaCl 0.9%). Particle mesh Ewald algorithm was applied with a cut-off radius of 8 Å. A timestep of 2.5 fs was set. The simulation snapshots were recorded at intervals of 100 ps until a total simulation time of 30 ns. Results were analyzed with a script included as part of YASARA software and included RMSD, ligand binding energy variations (using MM-PBSA calculations), and distance of ligand 87 to Ser 283 (*Dm*AChE) or Ser 203 (*h*AChE), as these residues play a key role in acetylcholinesterase enzymatic activity. For *Dm*AChE, we considered the interaction between the primary alcohol group of the central glucopyranose ring with Ser 238, and for *h*AChE, the interaction between the hydroxyl group of the ferouyl residue with Ser 203. A similar procedure has been recently reported for the simulation of complexes of drugs with some proteins [41,42,43].

### 2.4. Construction of the Virtual Multitarget Index and the Weighed Multi-Target Index

The virtual Multitarget index of each compound was determined. To compare the multitarget index of the analyzed compounds, we propose a virtual multitarget index (*vMTi*), which was calculated for the three insect targets (EcR, PPO, and *Dm*AChE) using formula (1):

(1) $$vMTi=\sum_{i=1}^{n}\frac{MD_{i}}{MDr}$$

where MDi corresponds to the *MolDock* score of the molecule in a specific target and MDr is the *MolDock* score of the reference ligand; we considered the compound with the lowest *MolDock* score (which has the highest theoretical affinity) for each target as the reference. Compounds with higher values have a higher multitarget index. However, binding to *h*AChE is an undesirable condition. To take this into consideration, we propose a weighed *MTi* (*wMTi*), which was calculated using formula 2, using an external coefficient *n*, which represents the desirability of binding to a specific target:

(2) $$wMTi=\sum_{i=1}^{n}n\frac{MD_{i}}{MDr}$$

To calculate *wMTi*, we gave values of *n* = 0.3 to desirable targets (EcR, PPO, and *Dm*AChE) and *n* = −0.3 to *h*AChE.

In addition to these calculations, a contour plot was built with Minitab using PPO, *Dm*AChE, and EcR docking scores. This plot can help identify potential multitarget compounds because those compounds would appear in valleys since they would have lower *MolDock* scores.

## 3. Results

### 3.1. Docking Studies Results

Table 1, Table 2 and Table 3 show data for the 10 compounds with a lower average *MolDock score* in the ecdysone receptor (EcR), prophenoloxidase (PPO), and acetylcholinesterase (both *Dm*AchE and *h*AChE) docking study. A table with complete docking results is presented in Supplementary Information.

### 3.2. Molecular Dynamics Studies on Complexes of Verbascoside with DmAChE and hAChE

**Figure 1:**
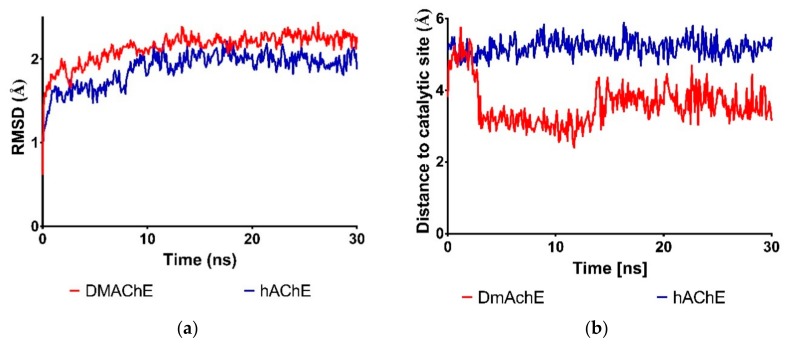
Plots of variations along time of Molecular Dynamics (MD) simulations of complexes of **87** with *Dm*AChE (red) and *h*AChE (blue). (**a**) The Root Mean Square Deviation (RMSD) of protein backbone; (**b**) distance of compound **87** to Ser 238 (*Dm*AChE) or Ser 203 (*h*AChE).

Figure 1a shows the comparison of the RMSD time profile for protein backbone atoms during the 30 ns simulation of the complexes of compound **87** and *Dm*AChE and *h*AChE. Both complexes have RMSD average values around 2 Å (RMSD = 2.13 Å for *Dm*AChE and RMSD = 1.88 Å for *h*AChE).

We also wanted to check if the distance of compound **87** (verbascoside) to the catalytic site of acetylcholinesterase variates during the simulation time. We selected the potential interactions to Ser 203 in *h*AChE or Ser 238 in *Dm*AChE predicted by molecular docking as described in Methodology. Figure 1b shows the variation of distances of compound **87** to these key serine residues along the simulation time. As seen in this figure, the distance to catalytic site diminished from 4.8 Å to 3.0 Å after 3 ns of simulation in the case of *Dm*AChE (average distance = 3.6 Å), while it maintained almost the same in *h*AChE (average distance = 5.21 Å).

To estimate the difference in binding energy of compound **87** in its complex with *Dm*AChE and *h*AChE, MM-PBSA methods were applied. Ligand binding energy suggests better binding of compound **87** to *Dm*AChE (E = −131.5 kJ/mol) versus *h*AChE (E = −134.0 kJ/mol) as binding energy calculations implemented in YASARA Dynamics indicates that the higher the energy value, the better the binding.

### 3.3. Construction of the Virtual Multitarget Index and the Weighed Multitarget Index

**Figure 2:**
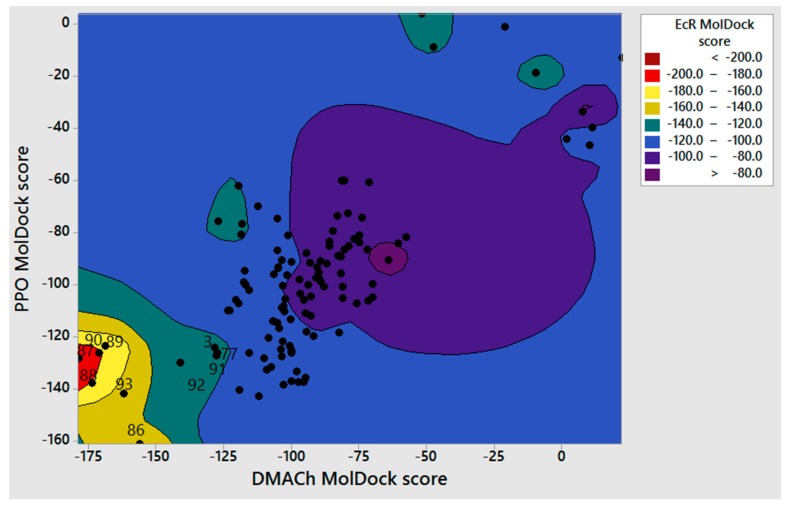
Contour plot correlating PPO, *Dm*AChE, and EcR docking scores. Zones in red-yellow indicate higher affinity to EcR than zones in purple or blue.

**Figure 3:**
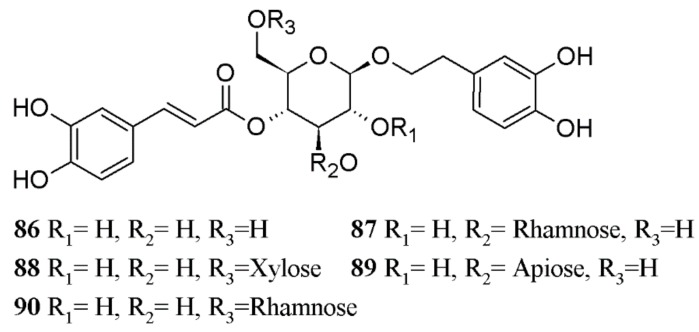
Structures of phenylethanoid glycosides (compounds **86**–**90**) which exhibited the highest *vMTi* and *wMTi* values.

Figure 2 shows a contour graphic that compares docking scores of the analyzed compounds on PPO, *Dm*AChE, and EcR. The zone in red corresponds to those molecules with high theoretical affinity against all the three molecular targets. Figure 3 shows the structure of the phenylpropanoids which resulted with the highest values of *vMTi* and *wMTi*. Table 4 shows a list of compounds with higher *vMti* and *wMTi* values; a full list is included in Supplementary Information.

## 4. Discussion

### 4.1. Docking Studies on Ecdysone Receptor

Induction of molting in Arthropods coincides with a release of 20-hydroxyecdysone (20-E), a steroidal-type hormone. Prior to each of the larval molts, at pupariation, at pupation, and during metamorphosis, the hormone is released in carefully timed spurts, coinciding with major morphological transitions. Ecdysone receptor (EcR) exists in three isoforms. Each requires a partner during the heterodimerization, a *Drosophila* homolog of vertebrate RXR protein named ultraspiracle (USP) protein. Although ecdysone can bind to EcR on its own, binding is significantly augmented by the participation of USP. The interaction between EcR and ecdysone is a crucial event for the development of insects, which is why it represents an interesting molecular target against pest insects [44].

**Figure 4:**
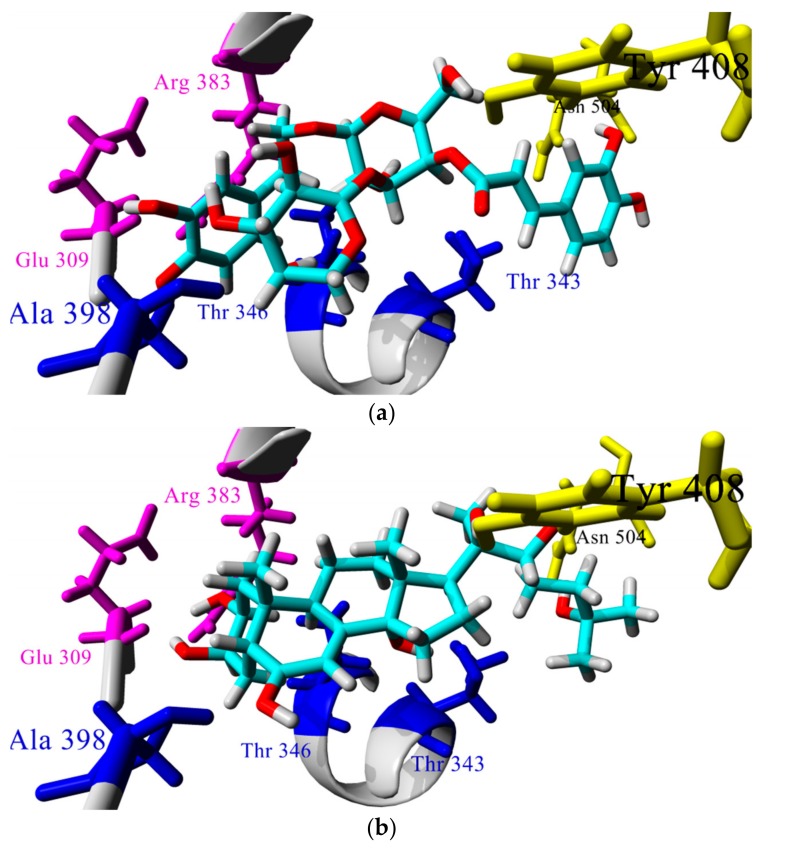
Comparison of (**a**) the docked pose of verbascoside (compound **87**) and (**b**) 20-E crystallized in the LBD of EcR (PDB:2R40).

Table 1 shows data for the 10 compounds with a lower average *MolDock score* in the ecdysone receptor docking study. Phenylethanoid glycoside derivatives have better theoretical binding to EcR among the evaluated compounds. Among them, the results indicate that binding to EcR occurs through hydroxyl groups of caffeoyl and phenyl ethyl residues in similar fashion to 20-E (Figure 4a). Analysis of Figure 4a,b reveals that compound **87** (verbascoside, which is a major phenylethanoid glycoside present in *Calceolaria* extracts) adopted a J-shaped conformation in the binding site of EcR, which is similar to the conformation adopted by natural ligand of EcR, 20-E [31] and the interaction pattern is very similar between these compounds: phenylethanoid residue interacts through hydrogen bonds with Arg383 and Glu309 (in magenta in Figure 4a,b) like 2β and 3β hydroxyl groups of ring-A in 20-E, rhamnose residue interacts with Ala398 (in blue in Figure 3a,b) like C-6 ketone moiety in ring-B in 20-E, feruoyl residue interacts with Thr 343 and rhamnose residue with Thr 346 (also in blue in set of Figure 4) like 14-α hydroxyl group, and additional interactions between feruoyl residue and central glucose ring of verbascoside with Tyr 408 and Asn 504 (shown in yellow) are seen in a similar fashion for 25-OH group of side chain of 20-E. This interaction is notable because 20-E interacts with this residue via a water linkage [31], hence the interaction of verbascoside with Asn504 could increase affinity, as some studies have demonstrated that ligands designed to displace the water molecules exhibit higher affinity [45].

Though there are no previous studies on phenylethanoid glycosides as EcR ligands, there is some experimental evidence that can support docking results. It has been previously reported that the ethyl acetate extract of *C. talcana*, of which verbascoside is its major component (compound **87**), caused a developmental disruption of *D. melanogaster* and *S. frugiperda* larvae. In addition, the authors of the study proposed verbascoside as a disruptor of ecdysteroid metabolism [26]. In another study, the incorporation of verbascoside in the artificial diet of the pest *Agrilus planipennis* caused a 100% mortality at 45 mg/g of artificial diet [46] when testing the toxic effect of verbascoside against at least three different pest insect species. In addition, Harmatha and Dinan have reported that some polyhydroxylated stilbenoids have an antagonist EcR activity [47], and a previously reported pharmacophore model indicated that the presence of hydroxyl groups in an ecdysteroid template is important for EcR binding [48]. On the other hand, the presence of some other phenylethanoid glycosides, such as calceolariosides A, B, and C, in active extracts of *Calceolaria* as well as in *Fraxinus* spp. can be related to the strong larval molting disruption observed when the larvae of different species were exposed to extracts with high amounts of phenylethanoid glycosides or directly to different amounts of the isolated compounds [46]. All these experimental data strongly suggest that phenylpropanoid glycosides could be EcR ligands.

As described here, the effect of verbascoside and related phenylethanoid glycosides against the ecdysone receptor can explain only one of the multiple effects exerted by these compounds. The experimental evidence remarkably indicates that molting disruption exerted by phenylethanoid glycosides cannot be the only mechanism that explains the strong exerted insecticidal properties. The above information suggests a possible antagonist and multienzymatic inhibitory mechanism that phenylethanoid glycosides can exert, causing larvae disruption activity by acting as EcR antagonist in addition to other mechanisms.

### 4.2. Docking Studies on Prophenoloxidase

Melanization, a process performed by phenoloxidase (PO) and controlled by the prophenoloxidase (PPO) activation cascade, plays an important role in the invertebrate immune system in allowing a rapid response to pathogen infection. The activation of the PPO system, by the specific recognition of microorganisms by pattern-recognition proteins (PRPs), triggers a serine proteinase cascade, which eventually leads to the cleavage of the inactive PPO to the active PO that functions to produce melanin and toxic reactive intermediates. The importance of PPO–PO is due to cuticular sclerotization and defense against pathogens and parasites. PO catalyzes hydroxylation of monophenols to o-diphenols and oxidation of o-diphenols to quinones. Quinones take part in sclerotization and tanning of the cuticle and serve as precursors for synthesis of melanin [49,50]. Therefore, PPO is a very suitable molecular target for designing pesticide compounds. 

Table 2 shows docking results from the PPO study. Among the compounds with better PPO binding, the best are flavonoids and phenylethanoid glycosides. The results are in agreement with several previous in vitro and in silico studies [10,51,52,53,54,55] in which most tyrosinase inhibitors possess a phenol moiety as the pharmacophore. Among them, flavonoids appear as effective competitive inhibitors of this enzyme. In addition, Karioti et al. [53] and Muñoz et al. [10] have reported tyrosinase inhibitory activities of some phenylethanoid glycosides, which have lower but comparable inhibitory activities compared with flavonols and flavones.

**Figure 5:**
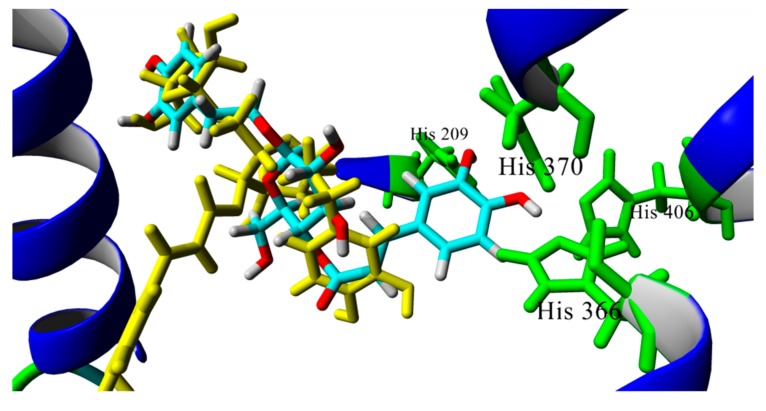
Overlap docking poses of compounds **86** (cyan) and **88** (yellow). Histidine residues of catalytic site are shown in green.

Hydroxyl groups of caffeoyl or phenylethyl residues have been proposed as essential structural requirements to display inhibitory activities against PPO because of their chelating properties. Figure 5 shows that phenylethanoid glycosides could bind to the PPO catalytic site through interaction with His residues, which are required to form a complex with copper ions. This is in agreement with the previously reported information that indicates verbascoside as a substrate for tyrosinase [10]. Among phenylethanoid glycosides, compounds **86** and **93** could bind better than other analogs. These compounds are monoglycosides, whereas other evaluated phenylethanoid glycosides are diglycosides, which is in agreement with previous reports that indicate the increase in the number of sugar units and the reduction of PPO inhibitory activity [53]. This observation could be explained in terms of the higher molecular volume of diglycosides that prevents them access to the active site as is shown Figure 5; the diglycoside compound **88** is shown in yellow and monoglycoside **86** is in cyan. From this figure, it can be concluded that it is possible that monoglycosides could bind closer to the catalytic site of PPO. 

### 4.3. Docking Studies and Molecular Dynamics Simulations on Drosophila and Human Acetylcholinesterase

Acetylcholinesterase is a serine hydrolase that is vital for regulating the neurotransmitter acetylcholine in insects. This enzyme is an excellent molecular target for the development of insecticides [56,57]. The well-known active site has a deep and narrow gorge, with a catalytic site at the bottom and a peripheral site at the entrance. Acetylcholinesterase is a molecular target used to control insects that affect public health (e.g., mosquitoes, flies, cockroaches, among others) as well as those that affect agriculture and gardening (e.g., grasshoppers, aphids, caterpillars, among others) [58]. Current anticholinesterase insecticides work through phosphorylation of a serine residue at the AChE catalytic site, which disables the catalytic function and causes enzyme antagonism. Because this serine residue is also ubiquitous in AChEs of mammals and other species with cholinergic nerves, the use of anticholinesterase insecticides to target the serine residue causes serious off-target toxicity [59]. Therefore, it is necessary to evaluate in silico the affinity of molecules against the insect together with mammalian enzymes to determine whether there are some structural features that lead to design inhibitors specifically against insect enzymes.

Table 3 displays the *MolDock* scores of the top 10 compounds with better average docking scores for *Dm*AChE studies in PDB 1DX4 and 1QON structures. Data obtained during docking studies with *h*AChE (PDB structures 4EY7 and 4M0E) is also shown. Selectivity ratio versus *h*AChE was calculated with the average docking score obtained in both enzymes. Although some differences could be appreciated in docking scores values, the same tendency was observed in both *Dm*AChE structures and both *h*AChE structures as phenylethanoid glycosides are among the compounds with a better theoretical binding in the four docking studies. It has been reported that verbascoside and extracts containing other phenylethanoid glycosides are moderate inhibitors of AchE [25]. 

**Figure 6:**
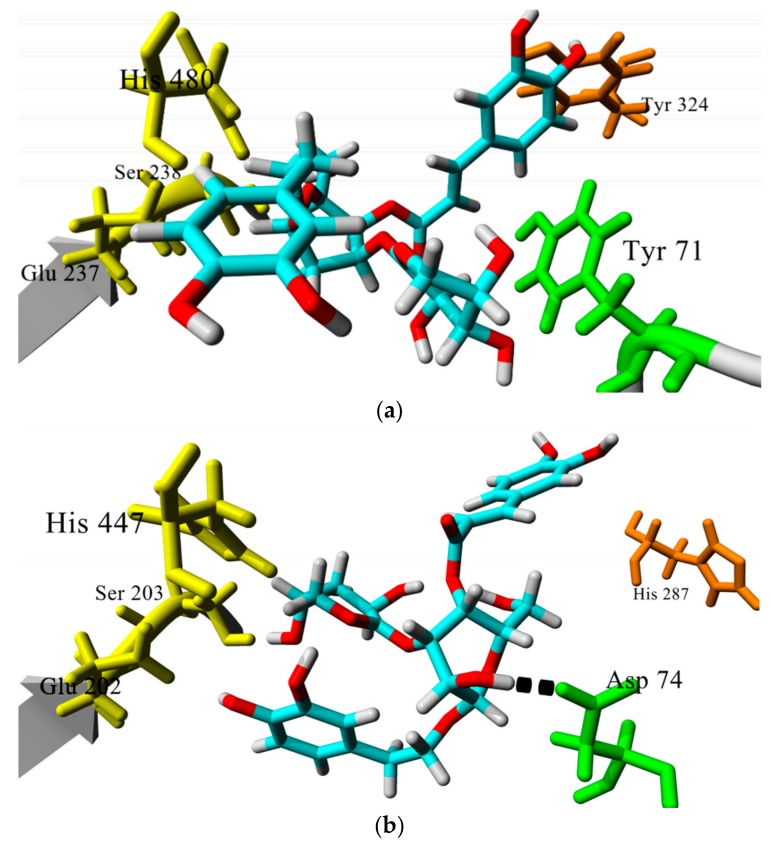
Comparison of the docking poses of compound **87** in *Dm*AChE (**a**) and *h*AChE (**b**). Catalytic residues are colored in yellow and residues at the entrance of the active site in orange. Tyr71/Asp 74 residues, which are different in each enzyme, are colored in green. Key hydrogen bond interaction of verbascoside to Asp 74 is shown in black.

Figure 6a,b shows the predicted binding mode of verbascoside (compound **87**) in the active site of both *Dm*AchE and *h*AChE, respectively. In the case of the docking study carried out in *Dm*AChE, verbascoside and the rest of the analyzed PEGs adopted a Y-shaped conformation, with the central sugar core interacting with residues of the catalytic triad (colored in yellow in Figure 6a) and the phenylethoxy chain interacting with other residues within the bottom of the gorge. In the case of *h*AChE, the analyzed PEGs adopted a similar conformation, but the phenylethoxy chain did not reach the bottom of the gorge. An explanation to this is that PEGs interact with Asp74 through a hydrogen bond in *h*AChE, whereas this residue is absent in *Dm*AChE [60], limiting the access of PEGs to the catalytic triad, and this could explain the better theoretical affinity of PEGs to *Dm*AChE compared to *h*AChE.

To analyze additional differences that could account for potential selectivity of PGs to *Dm*AChE over *h*AChE, MD simulations were performed. The RMSD values could indicate the stability of the protein relative to its conformation. Figure 4 shows the comparison of the RMSD of the protein backbone profile during the 30 ns simulation of the complexes of compound **87** and *Dm*AChE and *h*AChE. Both complexes have RMSD values around 2Å (RMSD = 2.13 for *Dm*AChE and RMSD = 1.88 for *h*AChE), suggesting that both complexes are stable.

An important difference that was observed during visual analysis MD simulations was the variation of distance of verbascoside (compound **87**) to catalytic site in *Dm*AChE, as it appeared to move closer to key residues Glu 237 and Ser 238, while it seemed that compound **87** maintained a constant distance to equivalent residues Glu 202 and Ser 203 in *h*AchE complex. We confirmed this by measuring the distance of verbascoside to Ser 238 or Ser 203. As seen in Figure 2b, the distance to catalytic site diminished from 4.8 Å to 3.0 Å after 3 ns of simulation in the case of *Dm*AChE (average distance = 3.6 Å), while it maintained almost the same in *h*AChE (average distance = 5.21 Å). This could account for the better binding of **87** towards *Dm*AChE. This could be an important difference that could be useful to future design of selective inhibitors to *Dm*AChE based on the phenylethanoid glycoside template. Additionally, MM-PBSA calculations suggest that 87 binds better to *Dm*AchE (E = −131.5 kJ/mol) than to *h*AChE (E = −134.0 kJ/mol). Overall, we conclude that, based on molecular docking calculations and MD simulations, compound **87** is an interesting starting point for the design of selective *Dm*AchE inhibitors, an important factor to consider in terms of potential toxicity against human beings.

### 4.4. Virtual Multitarget Index and Weighed Multitarget Index

A multitarget drug has been defined as the integration of multiple pharmacophores in one molecule with the purpose that it can have two or more simultaneous mechanisms of action [61]. Though the concept of multitarget drugs is an important research topic [62,63], there are few examples of the study of multitarget insecticides [12].

Computational tools like molecular docking and multitarget quantitative structure–activity relationship models (mt-QSAR) have been recently used for prediction and discovery of multitarget compounds [64,65,66,67]. However, the development of parameters to measure the multitarget index of a ligand is not an easy task. As described during the lapatinib discovery [68], the simple average of biological activity against several targets (or in our case, docking scores) can be misleading, because a compound with a promising average value of bioactivity on two or more targets could correspond to a multitarget compound or to a highly selective compound against only one target. Thus, a reference parameter and weighed coefficients for biological activities of interest should be included. Herein, we propose the use of contour graphics, such as the one shown in Figure 2, and a multitarget index (*vMTi*) to identify potential multitarget insecticides. In the contour plot of Figure 2, the docking scores in three targets of interest (EcR, PPO, and *Dm*AChE) are shown. Compounds that have shown greater theoretical affinity for the three targets will appear in the bottom left of the plot and inside the red and yellow contour areas. This would be a first criteria to identify potential multitarget compounds, because selective compounds would not appear in this area. As expected, the phenylethanoid glycosides appear in this area, but other compounds, such as abietane **3** and isopimarane **77**, can be considered to have potential multitarget properties.

Table 4 shows a list of compounds with higher *vMti* and *wMTi* values. In this table, phenylethanoid glycosides appear as compounds with a better multitarget profile. In addition, compounds **88**, **89**, and **87** (Figure 2) have not only high *vMTi* values, but also the highest *wMTi* due to their higher selectivity to *Dm*AChE than *h*AChE. Thus, these compounds are interesting candidates for the development and evaluation of safer multitarget insecticides.

## 5. Conclusions

Our results complement the experimental results obtained during *Calceolaria* extracts evaluation as biopesticides, and suggest that some of the compounds, such as the phenylethanoid family, can be used for the development of *multitarget* bioinsecticides. Based on the docking studies, it appears that verbascoside and other phenylethanoid glycosides could exert their bioactivity by modifying the activity of various receptors like EcR and enzymes like PPO and AChE, as was suggested and confirmed in previous experimental assays [25,26]. Theoretical affinity, together with *vMTi* and *wMTi*, can be useful for the rational design of *multitarget* bioinsecticides. Verbascoside appears as a good candidate for the development of a multitarget insecticide due to its prolific natural occurrence and its chemical and biological properties [69]. 

## Figures and Tables

**Table 1:** *MolDock Scores* obtained in the ecdysone receptor (EcR) docking. Top 10 compounds with the better theoretical binding. PEG = Phenylethanoid glycosides.

Ligand	Skeleton Type	Compound Name	PDB: 3IXP	PDB: 2R40	Average *MolDock Scores*
88	PEG	Calceolarioside C	−214.0	−207.2	−210.6
90	PEG	Forsythoside A	−202.8	−213.5	−208.2
89	PEG	Calceolarioside E	−184.7	−212.5	−198.6
92	PEG	Isoarenarioside	−183.0	−205.5	−194.2
87	PEG	Verbascoside	−197.4	−184.1	−190.8
86	PEG	Calceolarioside A	−174.2	−183.6	−178.9
91	PEG	Calceolarioside B	−162.1	−176.7	−169.4
93	PEG	Calceolarioside D	−160.2	−169.3	−164.7
68	Scopadulane	3-Isovaleroyl-7-malonyloxy-thyrsiflorane	−147.2	−157.1	−152.2
45	Isopimarane	3-β-Isovaleroyl-18-hydroxy-7-α-malonyloxyent-isopimara-9(11), 15-diene	−150.2	−151.5	−150.8

**Table 2:** *MolDock Scores* obtained in the PPO docking. Top 10 compounds with the better theoretical binding. PEG = Phenylethanoid glycosides.

Ligand	Skeleton	Compound Name	*MolDock* Score
86	PEG	Calceolarioside A	−161.187
110	Flavonoid	Kaempferol-7-methyl ether	−142.825
93	PEG	Calceolarioside D	−142.017
109	Flavonoid	Gossypetin-7,8,3′-trimethyl ether	−140.618
108	Flavonoid	Herbacetin-8,4′-dimethyl ether	−138.595
88	PEG	Calceolarioside C	−137.969
111	Flavonoid	Kaempferol-4′-methyl ether	−137.519
104	Flavonoid	Naringenin-4′-methyl ether	−137.451
107	Flavonoid	Isoscutellarein-8,4′-dimethyl ether	−137.188

**Table 3:** *MolDock* scores obtained in the AChE docking study. Top 10 compounds with the better theoretical binding.

		*Dm*AChE *MolDock* Scores			*h*AChE *MolDock* Scores			
Ligand	Compound Name	PDB: 1DX4	PDB: 1QON	Average Score	PDB: 4EY7	PDB: 4M0E	Average Score	SR ^1^
90	Forsythoside A	−171.3	−254.5	−212.9	−177.7	−247.6	−212.6	1.00
88	Calceolarioside C	−174.0	−251.6	−212.8	−145.7	−217.7	−181.7	1.17
87	Verbascoside	−178.8	−233.5	−206.1	−152.6	−200.8	−176.7	1.17
89	Calceolarioside E	−169.0	−239.1	−204.0	−116.7	−209.0	−162.8	1.25
93	Calceolarioside D	−162.3	−227.7	−195.0	−147.9	−188.4	−168.2	1.16
92	Isoarenarioside	−141.1	−244.8	−193.0	−165.1	−208.0	−186.6	1.03
86	Calceolarioside A	−156.2	−212.8	−184.5	−164.6	−189.6	−177.1	1.04
91	Calceolarioside B	−128.0	−210.5	−169.2	−137.9	−183.9	−160.9	1.05
44	Isopimarane	−119.9	−180.3	−150.1	−137.0	−159.7	−148.4	1.01
43	Isopimarane	−127.3	−164.5	−145.9	−150.2	−154.6	−152.4	0.96

^1^ Selectivity ratio (SR) = Average docking score *Dm*AChE/Average docking score *h*AChE.

**Table 4:** Compounds with higher *vMTi* and *wMTi* values. PEG = Phenylethanoid glycosides.

Ligand	Skeleton	Compound Name	*vMTi*	*wMTi*
88	PEG	Calceolarioside C	2.86	0.60
89	PEG	Calceolarioside E	2.67	0.57
86	PEG	Calceolarioside A	2.72	0.56
87	PEG	Verbascoside	2.67	0.55
93	PEG	Calceolarioside D	2.58	0.54
90	PEG	Forsythoside A	2.77	0.53
92	PEG	Isoarenarioside	2.64	0.53
91	PEG	Calceolarioside B	2.39	0.49
109	Flavonoid	Gossypetin-7,8,3′-trimethyl ether	2.05	0.43
110	Flavonoid	Kaempferol-7-methyl ether	2.03	0.43
77	Labdane	19-Malonyloxy-9-epi-ent-labda- 8(17), 12 Z, 14-triene	2.04	0.42
45	Isopimarane	3-β-Isovaleroyl-18-hydroxy-7-α-malonyloxyent-isopimara-9(11), 15-diene	2.13	0.42
3	Abietane	19-Malonyloxy-dehydroabietinol	1.99	0.42
57	Stemarane	17-Acetoxy-19-malonyloxy-ent-stemar-13(14)-ene	2.03	0.41
108	Flavonoid	Herbacetin-8,4′-dimethyl ether	1.92	0.40

## Supplementary Materials

The following are available online at http://www.mdpi.com/2218-273X/8/4/121/s1, Table S1: *MolDock* scores and multitarget index for all analyzed compounds, Table S2: MD simulation data.
